# Developing a scalable model of recombinant protein yield from *Pichia pastoris*: the influence of culture conditions, biomass and induction regime

**DOI:** 10.1186/1475-2859-8-35

**Published:** 2009-07-01

**Authors:** William J Holmes, Richard AJ Darby, Martin DB Wilks, Rodney Smith, Roslyn M Bill

**Affiliations:** 1 School of Life and Health Sciences, Aston University, Aston Triangle, Birmingham, B4 7ET, UK; 2 Alpha Biologics UK Ltd Building 201, Babraham Research Campus, Babraham, Cambridge, CB22 3AT, UK; 3 Cameron International Ltd, Queen Street, Stourton, Leeds, LS10 1SB, UK

## Abstract

**Background:**

The optimisation and scale-up of process conditions leading to high yields of recombinant proteins is an enduring bottleneck in the post-genomic sciences. Typical experiments rely on varying selected parameters through repeated rounds of trial-and-error optimisation. To rationalise this, several groups have recently adopted the 'design of experiments' (DoE) approach frequently used in industry. Studies have focused on parameters such as medium composition, nutrient feed rates and induction of expression in shake flasks or bioreactors, as well as oxygen transfer rates in micro-well plates. In this study we wanted to generate a predictive model that described small-scale screens and to test its scalability to bioreactors.

**Results:**

Here we demonstrate how the use of a DoE approach in a multi-well mini-bioreactor permitted the rapid establishment of high yielding production phase conditions that could be transferred to a 7 L bioreactor. Using green fluorescent protein secreted from *Pichia pastoris*, we derived a predictive model of protein yield as a function of the three most commonly-varied process parameters: temperature, pH and the percentage of dissolved oxygen in the culture medium. Importantly, when yield was normalised to culture volume and density, the model was scalable from mL to L working volumes. By increasing pre-induction biomass accumulation, model-predicted yields were further improved. Yield improvement was most significant, however, on varying the fed-batch induction regime to minimise methanol accumulation so that the productivity of the culture increased throughout the whole induction period. These findings suggest the importance of matching the rate of protein production with the host metabolism.

**Conclusion:**

We demonstrate how a rational, stepwise approach to recombinant protein production screens can reduce process development time.

## Background

Proteins lie at the heart of biology: understanding their structures and mechanisms of action gives direct insight into cellular function as well as providing targets for the investigation of disease. Since the vast majority of proteins are not sufficiently abundant from natural sources, recombinant overproduction is a universally-recognised solution to obtaining the milligram to gram quantities required for applications as diverse as structural genomics and biopharmaceutical manufacture [1]. Suitable host cell factories for producing recombinant proteins include microbes such as *Escherichia coli*, *Lactococcus lactis*, *Saccharomyces cerevisiae *and *Pichia pastoris*, as well as mammalian (e.g. CHO, NS0 and BHK cell lines) and insect cells (e.g. Sf9, Sf21, S2 and Tn5B1–4 cell lines) transfected with viral vectors. Each system has benefits and drawbacks in its use, but yeast with its well-established genetic and molecular biological resources combines the ease and speed-of-use of bacterial systems with its ability as a eukaryote to secrete post-translationally-modified proteins. As a consequence it is an increasingly popular choice in both academic and commercial laboratories [2,3]. Nonetheless the routine achievement of high production yields in yeast or indeed any of the alternative systems mentioned above continues to be a substantial bottleneck to further progress.

Following the initial identification of expressing clones, the methods to optimise and scale-up promising small-scale screens are still based on trial and error [4]. To rationalise this, several groups have recently adopted the 'design of experiments' (DoE) approach [5] frequently used in industry [6,7]. Studies have focused on how elements of the experimental set-up in bioreactors or shake flasks, such as medium composition [8], nutrient feed rates [9] and induction of expression [10] affect product yield. Recently Islam and colleagues [11] used DoE in a micro-well plate format to examine the optimisation of firefly luciferase production in *E. coli *by applying an experimental design that included liquid fill level and culture agitation rates as input parameters, both of which strongly influence oxygen transfer rates in the wells. The authors of this study found that while oxygen transfer was indeed important, they were only able to look at it indirectly in their set-up. In addition, pH which routinely varies in growth conditions unless controlled by a pH probe feedback loop or an appropriate buffer was not included as a factor in their DoE design. While the model (R^2 ^= 0.817) generated predicted values in good agreement with those obtained, the protein yields were unexpectedly low which appeared to result from lysed cells. Subsequently, the same authors examined how oxygenation efficiency (as measured by the oxygen mass transfer coefficient, *k*_*L*_*a*) is a parameter that can be used to guide scale-up from micro-well plate to bioreactor [12].

In this study we wanted to find a simpler, more direct method of scaling up promising small-scale production screens in yeast, thereby minimising trial and error. Here, we describe how a DoE-derived model generated using a parallel mini-bioreactor (that can directly control pH, temperature (T) and dissolved oxygen (DO) [13,14]) is predictive not only of protein yield normalised for both culture volume and density (termed here the 'specific yield') in the same system, but is scalable to *P. pastoris *cultures grown in a 7 L bioreactor. We also observed that the accumulation of pre-induction cell density in the batch phase and maximising the effective period of the fed-batch induction regime were critical to obtaining high total yields of functional protein by minimising methanol accumulation. Overall our approach should facilitate the stepwise scale-up of the best production conditions from bench to bioreactor.

## Methods

### Vectors

The *P. pastoris *expression vector was based on pPICZαA (Invitrogen Ltd.). GFP (cycle 3 mutant) cDNA originated from the pGFPuv vector (Clonetech Cat # 632312). PCR primers were designed to introduce *Eco*RI and *Xba*I restriction sites (bold) 5' and 3' respectively to the GFPuv coding sequence. The forward primer was 5' ACGT **GAA TTC **ATG AGT AAA GGA GAA GAA and the reverse primer was 5' TGCA **TCT AGA **GG TTT GTA GAG CTC ATC CAT. This allowed the insertion of GFPuv into the multiple cloning site of pPICZαA at *Eco*RI and *Xba*I, resulting in an expression cassette for secreted (α-factor) GFPuv with carboxy-terminal His_6 _and c-myc tags.

### Yeast strains and initial culturing conditions

*P. pastoris *strain X33 was denoted X33GFPuv following stable integration of *Pme*I-linearised pPICZαA-GFPuv. Positive clones were selected on YPDS agar with zeocin (100 μg mL^-1^). Shake flask cultures grown under standard handbook conditions of 30°C and 250 rpm agitation with BMGY/BMMY media [15] buffered to pH 6 with 100 mM potassium phosphate buffer, were used to confirm protein production. Supernatants were assayed as indicated below, and the highest yielding clone was selected for further study.

### Design of experiments and construction of the predictive model

**Figure 1:**
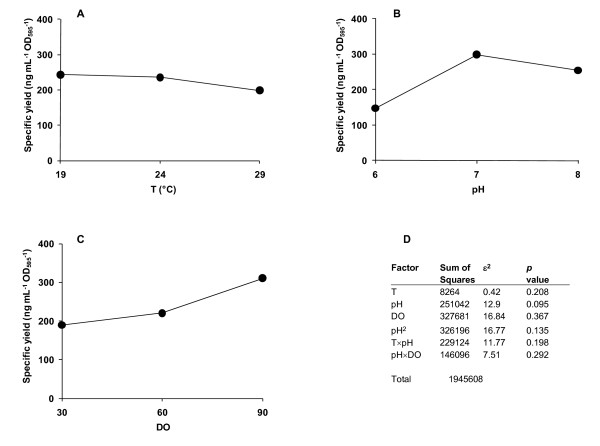
**Analysis of the model**. A main effects plot showing the influence of each of the input variables (A) T, (B) pH and (C) DO on specific yield. Panel D shows the ε^2 ^analysis which indicates the influence of each of the input factors and their interactions on the model. The value reported for ε^2 ^is the quotient of the sum of squares for the factor and the total sum of squares (from Table 2) expressed as a percentage.

**Figure 2:**
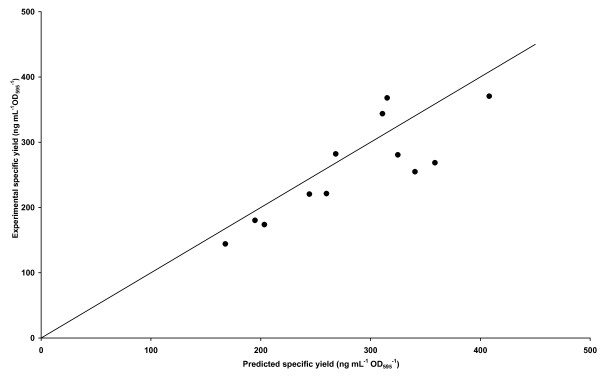
**Demonstration of the predictive capacity of the model**. A scatter plot of predicted versus experimental specific yield from M24 cultures is shown. Each check point condition was from within the model design space, but had not been used to build the model, as detailed in the Methods section. The fit to the line of parity (y = x) is shown with R^2 ^= 0.57.

Three factors which are typically varied in protein production experiments were investigated: pH, T and DO. Based on the results from an initial DoE (see additional file 1), these factors were each varied at three levels, coded as -1 (lowest value), 0 (middle value) and +1 (highest value) in a Box-Behnken design which uses a reduced number of experimental runs to generate a predictive model when compared to either full-factorial or central composite designs [16]. MiniTab statistical software (version 15.1.1.0) was used to construct the experimental matrix of factor combinations shown in Table 1. The predictive model generated from the outputs of the matrix is described in the Results section by Equation 1 and Figure 1. This final model was derived by removing terms from the full model based on their *p*-values, in descending order. The adjusted R^2 ^value (R^2^_adj_) for the regression changed as each term was removed, R^2^_adj _being a modification of R^2 ^that adjusts for the number of terms in the model [17]. R^2^_adj _values of 0.160 (full model), 0.115 (1 term removed), 0.274 (2 terms removed), 0.324 (3 terms removed) and 0.292 (4 terms removed) indicated that the model with 3 terms was statistically soundest. In Equation 1, the yield was converted to ng mL^-1^OD_595_^-1 ^from RFU mL^-1 ^OD_595_^-1 ^using an experimentally-derived factor, as described below. The results of the statistical validation of this model by ANOVA are shown in Table 2. The model was also validated experimentally by running the factor combinations shown in Table 3, which had not been used in the model building process, and comparing the fit of the experimental output to the predicted response from the model (Figure 2).

**Table 1:** Specification of the input factors and measurable outputs for the model building experiments.

INPUT FACTORS (controlled on-line)			MEASURABLE OUTPUT (measured offline)				
	
T (°C)	pH	DO (%)	OD_595_	RFU (mL^-1^)	Specific RFU (mL^-1 ^OD_595_^-1^)	Specific yield (ng mL^-1 ^OD_595_^-1^)	SD; n = 3 (ng mL^-1 ^OD_595_^-1^)
19	6	60	20.3	8651	426.2	127.9	3.2
19	8	60	0.8	1015	1268.8	380.6	3.9
19	7	30	13.1	10984	838.5	251.6	1.3
19	7	90	12.4	9259	746.7	224.0	2.1
24	6	30	24.4	8061	330.4	99.1	1.6
24	6	90	16.2	11951	737.7	221.3	5.6
24	8	30	4.7	1564	332.8	99.8	1.1
24	8	90	1.3	1954	1503.1	450.9	1.3
24	7	60	17.6	21382	1214.9	364.5	10.1
29	7	30	24.8	25392	1023.9	307.2	0.2
29	8	60	4.4	1413	321.1	96.3	1.5
29	6	60	21.7	10349	476.9	143.1	0.3
29	7	90	15.1	17495	1158.6	347.6	3.5

The input factors were temperature (T), pH and % dissolved oxygen (DO). Relative fluorescent units (RFU) and the optical density at 595 nm (OD_595_) were measured in triplicate 48 h post induction. The mean values are reported for 1 mL of culture. The standard deviation (SD; n = 3) is given for the specific yield of the M24 culture, where the conversion factor from RFU to ng was determined by generating a standard curve as described in the Methods section. All experiments were performed in batch in the M24 in BMMY medium.

**Table 2:** Statistical significance of the predictive model by analysis of variance (ANOVA).

Source	Degrees of Freedom	Sum of Squares	Mean Square	*F *statistic	*p *value
Regression	6	1288405	214734	1.96	0.217
Linear	3	586988	223083	2.04	0.21
Square	1	326196	326196	2.98	0.135
Interaction	2	375221	187610	1.71	0.258
Residual	6	657203	109534		

Total	12	1945608			

The statistical significance of the relationship between the predictors and the response of the model was assessed using analysis of variance (ANOVA), which employs Fisher's *F*-test. The goodness of fit of the model is 66%, as determined by the quotient of residual sum of squares/total sum of squares (R^2 ^= 0.66).

**Table 3:** Specification of the input factors for the model validation experiments.

T(°C)	pH	DO(%)
20	7.5	60
20	7.7	80
27	8	50
28	7.5	90
28	6	80
23.6	7.25	60
27.5	6.7	80
27.5	6.5	60
27.5	6.3	60
21.5	7.6	20
21.5	7.6	40
21.5	7.6	60

The input factors were temperature (T), pH and dissolved oxygen (DO)

### M24 set-up

The M24 system (Applikon Biotechnology Ltd) uses a disposable incubation cassette (a modified deep 24-well plate) with each well individually monitored and controlled for T, pH and DO [13]. Briefly, pH and DO conditions are monitored *via *optical sensors in the bottom of the wells and adjusted by sparging gases (O_2_, CO_2 _and NH_3 _vapour) through the cultures. T is controlled and monitored by a thermocycler-style device which is integral to the shaking platform. The wells of the incubation cassette have individual vented closures. Here we used type D closures, which have a one-way valve to prevent evaporation. For model-building experiments, conditions for growth (glycerol carbon source; BMGY) and production (methanol carbon source: BMMY) were defined as separate phases. Standard growth conditions for biomass accumulation were in 50 mL BMGY medium grown at 30°C, pH 6.0, 250 rpm with Fluka polypropylene glycol P2000 (Sigma Aldrich Cat # 81380) antifoam at 700 ppm in 500 mL baffled shake flasks. Production conditions in the M24 were according to the DoE matrix (Table 1) or the model validation conditions (Table 3). Where pH conditions differed from pH 6.0, the phosphate buffer component of BMGY/BMMY was altered accordingly. Agitation was set at 800 rpm and T, pH and DO set-points were defined according to Tables 1 or 3. Each well contained 6 mL BMMY buffered to the required pH with potassium phosphate buffer and 3.5 μL P2000 antifoam. Wells were inoculated to a target OD_595 _of 1.0 using the BMGY shake flask cultures detailed above. Induction was maintained by aseptically adding sterile methanol to 1% culture volume at 24 h post-induction. We noted that it was preferable to add the MeOH, and then work in a closed system to avoid evaporation, analogous to standard procedures in shake flasks. Growth was monitored by off-line OD_595 _measurements and product yield was measured by off-line fluorimetry 48 h post induction.

### Fed-batch bioreactor cultures

A saturated 20 mL BMGY shake flask-grown culture was used to inoculate a 200 mL BMGY seed culture in a 2 L baffled shake flask to a target OD_595 _of 1.0. This was grown to an OD_595 _of 2–15 and used to inoculate either a) 3 L BMGY containing 2 mL P2000 antifoam equilibrated to 30°C, pH 6.0, with a 30% DO set point in a 7 L (total volume) jacketed glass bioreactor with 3 × 6-blade Rushton impellars or b) 1 L BMGY containing 0.5 mL Antifoam A (Sigma-Aldrich) equilibrated as above in a 2.5 L (total volume) jacketed glass bioreactor (Applikon Biotechnology Ltd). The maximum gas flow rates used were 2 vvm air and 1 vvm O_2_. The maximum agitation rate was 1000 rpm. The glycerol batch phase was continued until all glycerol had been consumed, indicated by a spike in DO to 100%. A starvation phase was maintained for 1 h followed by an adaptation phase during which set-points were changed over 1 h to induction conditions as shown in Table 4. Once the 3 L cultures were at induction set-point, a standard 48 h 100% v/v methanol (0.2 μm filter-sterilised methanol with 12 mL L^-1 ^PTM1 trace salts) fed-batch induction phase was started with an initial flow rate of 0.167 mL min^-1 ^for 2 h, 0.417 mL min^-1 ^for 2 h and a final rate of 0.583 mL min^-1^. Alternatively, a mixed feed induction used 60% w/v D-sorbitol, 40% v/v methanol with 12 mL L^-1 ^PTM1 trace salts and a constant feed rate of 0.167 mL min^-1^. Vessel control was *via *an Applikon ADI1030 control unit. For the 1 L cultures, standard methanol (100% v/v, 20% v/v or 10% v/v; 0.2 μm filter-sterilised with 12 mL L^-1 ^PTM1 trace salts) or mixed feed (60% w/v D-sorbitol, 20% v/v methanol with 12 mL L^-1 ^PTM1 trace salts) fed-batch induction phases were started with initial flow rates of 4 mL h^-1 ^for at least 4 h, 8 mL h^-1 ^for at least 4 h and a final rate of 10 mL h^-1^. Vessel control was *via *an Applikon ADI1010 control unit.

**Table 4:** Conditions of the bioreactor adaptation phase under optimal DoE conditions.

Time (min)	T (°C)	pH	DO (%)
0	30	6	30
15	28	6.4	45
30	25	6.8	60
45	23	7.2	75
60	21.5	7.6	90
75	21.5	7.6	90

The input factors, T (°C), pH and DO (%) were adjusted every 15 min over the course of 1 h via an ADI1030 controller. Once optimal DoE conditions (21.5°C, pH 7.6, 90% DO) were reached, a further 15 min stabilisation period was applied prior to fed-batch induction.

### Sampling, extracellular substrate determination and fluorescence measurements

A harvest point 48 h post induction was selected following preliminary shake flask studies. Samples were withdrawn at various points throughout the growth curve to monitor GFP content. GFP concentration was determined using a Bio-Rad (Hemel Hempstead, UK) Bradford-based assay with bovine serum albumin as standard. Culture supernatant samples were taken and prepared for ESI-QUAD-TOF mass spectrometry allowing confirmation of the 28 kDa band on SDS-PAGE as GFP. Culture supernatants (100 μL) were additionally assayed using a fluorescent plate reader (SpectraMax Gemini or PerkinElmer Victor3 with a GFP specific filter set) with an excitation wavelength of 397 nm and an emission wavelength of 506 nm. All samples and blanks were buffered to pH >7.0 using 50 μL 1 M potassium phosphate pH 8.0. Methanol analysis was performed on a Unicam 610 GLC Gas Chromatograph. Appropriately-diluted culture supernatants (1 μL) were injected in duplicate and the methanol peaks integrated using ProGC software. The mean value of the integrated peak area was used to estimate the residual methanol within the sample by comparison with freshly-prepared calibration curves.

### SDS-PAGE and yield analysis

12 μL supernatant were loaded per lane on a NuPAGE 4–12% Bis-Tris polyacrylamide gel (Invitrogen Ltd. Cat # NP0322BOX) and separated by SDS-PAGE at 180 V for 45 minutes. Immunoblotting was carried out using 1:5,000 dilution of rabbit polyclonal anti-His_6 _antibody conjugated to HRP (Abcam Cat # ab1187-100) and visualised using EZ-ECL reagent (Biological Industries Cat # 20-500-120). The linear relationship RFU = 3.101 × 10^-7 ^mg GFP (R^2 ^= 0.93; data not shown) was confirmed between the density of the 28 kDa protein band (GFPuv) on a Coomassie-stained SDS-PAGE gel and relative fluorescence (RFU) of the same sample. This allowed the use of fluorescence as a quick and simple method of yield determination.

## Results

A typical profile of a *P. pastoris *culture producing a recombinant protein consists of glycerol batch and fed-batch phases, where biomass is accumulated; a starvation phase where all remaining glycerol is utilised; an adaptation phase which allows process conditions to be gradually changed from those for optimal cell growth to those for optimal protein production; and finally induction phases where the inducer (typically methanol) is supplied to the cells. In this study, we wanted to investigate the influence of the three most commonly-varied input parameters (T, pH and DO) on recombinant protein yield and therefore designed experiments to specifically optimise the induction phase separately from the biomass accumulation phase. In order to achieve this in an efficient manner, we used a DoE-generated matrix and a parallel mini-bioreactor that can control pH, T and DO.

### A predictive model of the induction phase can be generated to describe secreted recombinant GFP yield as a function of pH, T and DO in microwell format

We used a classic quadratic Box-Behnken design [16], which allows a reduced number of treatment combinations to be used in building the model and gives no bias towards any potential optimum region of the process space. All experiments were performed in a M24 parallel multi-well bioreactor (Applikon Biotechnology Ltd). This set-up provided on-line control of pH, T and DO in a 24 well plate format, each well having a working volume of 6 mL; 60% of the total volume. We defined our experimental set-up in the M24 to be 48 h in duration and reasoned that final yield and final biomass were key read-outs when maximising the total yield of a protein production experiment.

A first DoE (detailed in additional file 1) did not yield a model that described the process conditions associated with maximum yield. The data suggested that the design space of a follow-up DoE should examine relatively high pH and relatively low T. The values selected for this experiment were governed by both the technical specification of the M24 and the biology of *P. pastoris*. Table 1 summarises the input variables and measured outputs for the model building experiments which were consequently performed in the second, follow-up DoE presented here.

On examining the cultures, we noted that the overall relationship between total yield and the growth of the cultures (as measured by OD_595_) was found to fit to the line y = 1.31 × (R^2 ^= 0.53; data not shown), suggesting that optimal growth is not necessarily correlated to optimal protein production, as we [18] and others [19] have previously reported. Culture pH had a major influence on cell density, with the highest values being achieved at pH 6.0 (mean OD_595 _20.7, Table 1) and the lowest at pH 8.0 (mean OD_595 _2.8), while the effect of temperature and DO on growth were less marked, again as previously described [20].

We hypothesised that a model of recombinant protein yield normalised to both culture volume and density ('specific yield') as a function of T, pH and DO should be predictive over a range of different culture set-ups. Moreover, such a model might allow a two step optimisation, allowing for specific accumulation of biomass prior to induction using the predicted optimal production conditions of the model, thus leading to further improvement in total yield. Using the data in Table 1, a model was therefore constructed (Equation 1) as outlined in the Methods section, which described the relationship between specific yield and all input parameters.

**Equation 1: **Yield (ng mL^-1 ^OD_595_^-1^) = (- 21814.9 + (328.6 × T) + (5502.1 × pH) - (37.8 × DO) - (325.6 × pH^2^) - (47.9 × T × pH) + (6.4 × pH × DO)) × γ, where; T = temperature (°C), DO = dissolved oxygen (%) and γ = 0.3 and is the conversion factor from RFU to ng of protein, as described in the Methods section.

Figure 1 summarises the main effects plot for each input parameter on the specific yield. In agreement with the first DoE (additional file 1), yields improved at lower T and higher pH, although at the temperatures tested T did not have a large effect on yield (Figure 1A), which was highest around pH 7 (Figure 1B). Yields also increased with increasing DO (Figure 1C). Figure 1D shows the ε^2 ^results [21], which indicate the influence of each of the factors and their interactions within Equation 1. The data support the view that pH is a key factor as the ε^2 ^values for pH, pH^2 ^and the interactions of pH with both T and DO are substantial. DO alone is also important, while in contrast the effect of T alone makes a relatively small contribution, in agreement with the main effects plots (Figure 1).

Table 2 summarises the statistical assessment of the model by analysis of variance (ANOVA). Regression analysis of the model indicated that it explains 66% of the response variation. A recent report suggests that this type of analysis is often missing in published models and that good models from the literature have R^2 ^values > 0.75 with values below 0.25 being considered poor [5]. This suggested that our model was of acceptable quality in line with recent DoE studies of protein production in *E. coli *[5].

To validate the experimental quality of our model, predicted specific yields of GFP that were not used in the model building process were experimentally determined in the M24 and compared (Figure 2). Figure 2 shows the correlation between the predicted and measured specific yields. These experimental data fit to the line of parity with an R^2 ^= 0.57 suggesting that the predictive capacity of the model is acceptable. Nine of the twelve data points were within 40 ng mL^-1 ^OD_595_^-1 ^(i.e. within 5–15%) of the predicted value. The three data points outside this range (with T, pH, DO values of 20, 7.5, 60; 28, 7.5, 90 and 27.5, 6.7, 80), were within 16–25% of the predicted value, and were not correlated in any obvious manner.

### The model is scalable to a 7 L bioreactor

**Figure 3:**
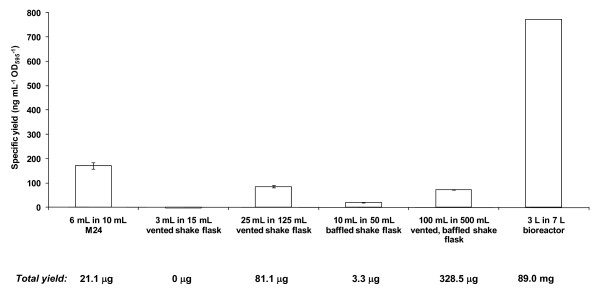
**Examination of the influence of culture vessel on GFP yield**. Shake flask cultures were set-up with a working volume of 20% total vessel volume, the M24 with 60% and the bioreactor with 43%. Vented cultures had a 0.2 μm integral filter as a closure. Non-vented cultures had a foil closure. Culture conditions were 30°C, pH 6 and either 250 rpm in the shake flasks, or DO 30% in the M24 and the 7 L bioreactor. The model predicts a specific yield of 219 ng mL^-1 ^OD_595_^-1^. Total yields for each condition are given.

We anticipated that the model (Equation 1) would not be predictive of specific yields from cultures grown in shake-flasks on account of their heterogeneous nature. To confirm this, different sized shake-flask cultures were induced by batch addition of methanol in line with commonly-used laboratory scale protocols for production screening (Figure 3). Under standard culture conditions (30°C, medium buffered at pH 6.0, culture volume set at 20% of the total volume and 250 rpm agitation), specific GFP yields were significantly different from that of 219 ng mL^-1 ^OD_595_^-1 ^predicted by the model for all cultures tested (Figure 3). This was in clear contrast with the results from the M24 mini-bioreactor (171 ng mL^-1 ^OD_595_^-1^; standard deviation 13 ng mL^-1 ^OD_595_^-1^) which were in excellent agreement with those predicted by the model (p = 0.004). We did note, however, that the total yield in shake flasks was directly related to culture size as described by the equation y = 3.25 × (R^2 ^= 0.99).

**Figure 4:**
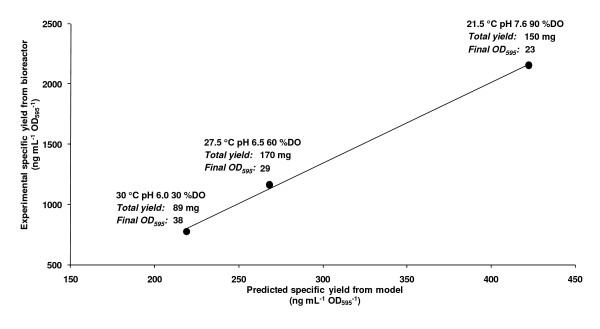
**Validation of the scalability of the model from 6 mL to 3 L working volume**. Three sets of process conditions were scaled up in bioreactors; standard (30°C, pH 6.0, 30% DO), best experimental fit to the derived model (27.5°C, pH 6.5, 60% DO) and predicted optimal yield (21.5°C, pH 7.6, 90% DO). A scatter plot of predicted versus experimental yields are shown. The data fit to the line of best fit (y = 6.7016× - 669.41) with R^2 ^= 0.99. Under each point, the final OD_595 _of the culture and the total yield of GFP are given.

On scaling up to a fed-batch bioreactor under standard culture conditions with a standard fed-batch induction protocol [15], we noted that the model was not directly predictive of the specific yield (Figure 3). When we examined additional culture conditions, however, we noted that there was a linear relationship between the specific yields predicted by the M24-derived model and those obtained from the bioreactor (Figure 4) despite the differences in experimental set-up. The data fitted to y = 6.7016× - 669.41 with R^2 ^= 0.99 such that at 21.5°C, pH 7.6, 90% DO it was possible to more than double the specific yield over that obtained from standard *P. pastoris *conditions (30°C, pH 6.0, 30% DO).

### Increasing pre-induction biomass increases the total protein yield

**Figure 5:**
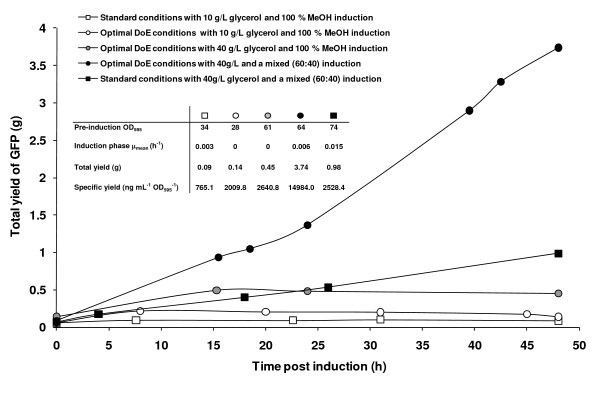
**Total GFP yields over a 48 h induction period in 3 L fed-batch cultures**. The effect of increased glycerol concentration in the medium and mixed feed induction regimes are demonstrated with standard (squares) and predicted optimal (circles) production conditions. The glycerol concentration was increased from 10 g L^-1 ^(white circles) to 40 g L^-1 ^(grey circles) prior to induction with methanol under DoE-predicted optimal conditions. Also shown are standard conditions (30°C, pH 6.0, 30% DO, methanol induction; white squares), for comparison. Black symbols indicate that the culture was grown with both 40 g L^-1 ^glycerol and a mixed 60% methanol, 40% sorbitol induction regime. Inset are data for pre-induction OD_595_, mean specific growth rate (μ_mean_; h^-1^), total and specific yields to allow comparison of the cultures.

**Figure 6:**
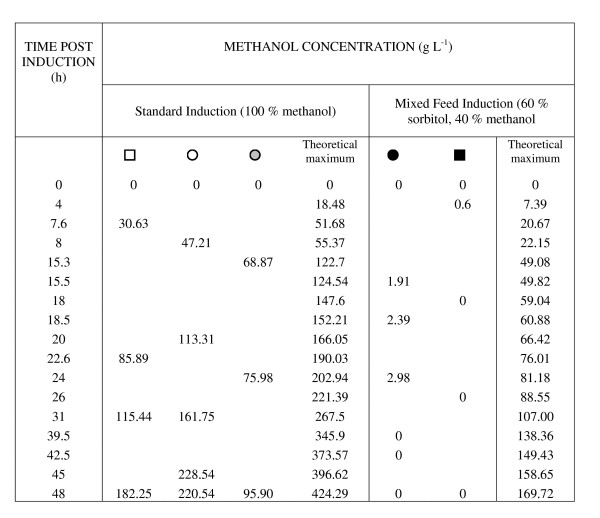
**Analysis of residual methanol concentrations from cultures in Figure 5**. Residual methanol concentrations are shown in g/L. The theoretical maximum methanol concentration (if none had been metabolised) is presented for comparison. The symbols used in Figure 5 are used here to identify standard (squares) or predicted optimal (circles) production conditions. Glycerol concentrations of 10 g L^-1 ^(white circles) and 40 g L^-1 ^(grey circles) prior to induction with methanol under DoE-predicted optimal conditions are also shown. Standard conditions (white squares) are given for comparison. Black symbols indicate that the culture was grown with both 40 g L^-1 ^glycerol and a mixed methanol/sorbitol induction regime.

Although the specific yield was maximal under optimal DoE conditions, the total yield was not on account of a lower final biomass (Figure 4). We therefore examined whether increasing the pre-induction culture biomass might improve the total yield in the culture. On transferring from the M24 to bioreactors, we had used Invitrogen's *P. pastoris *BMGY medium for ease of comparison, and induced the cultures using standard fed-batch induction conditions with 100% methanol. BMGY contains 1% glycerol, which is a factor of 4 less than the glycerol concentration used in typical basal salts medium (Invitrogen), which itself was unsuitable for our purposes as it precipitates heavily above pH 6.0 [22]. When we supplemented BMGY with glycerol to a final concentration of 4%, we found a doubling in OD_595 _on going from 1% to 4% glycerol (Figure 5). White circles denote cells growing on 1% glycerol under the optimal DoE conditions of 21.5°C, pH 7.6, 90% DO, while grey circles denote cells on 4% glycerol as carbon source under the same culture conditions. This resulted in a total yield improvement by a factor of 3 (from 140 mg to 450 mg GFP). The corresponding methanol data (Figure 6) suggest that this is because there is less (approximately half as much throughout the growth curve) residual methanol when the culture has an increased pre-induction biomass.

### Altering the induction strategy further improves yield

On inspection of the production phase of our cultures when induced with 100% methanol, we noted that methanol accumulation was occurring 8–15 h post induction, depending on the culture conditions. Not surprisingly, this accumulation was higher for cells growing at 21.5°C (white circles) than for those at 30°C (white squares) on account of their presumed slower cellular metabolism, and hence a poorer match with the standard induction conditions. While it was not our intention to perform a comprehensive analysis of the effect of different induction conditions on our model, we sought to demonstrate that matching the induction regime to the precise culture conditions might have additional benefits beyond the improvements conferred by the culture parameters themselves. A previously-validated mixed feed induction strategy [23,24] was therefore investigated using 60% w/v D-sorbitol, 40% v/v methanol at the lowest addition rate used in the standard induction conditions (0.167 mL min^-1^) for 48 h. We anticipated that this might be effective in two ways: first the concentration of methanol supplied to the culture is reduced due to dilution with sorbitol and second the non-repressive carbon source, sorbitol, provides an additional carbon source for biomass generation.

Figure 5 demonstrates how moving from a standard methanol induction to a mixed feed induction for cells cultured on 4% glycerol improved the total yield by a factor of 11 at standard conditions (white squares to black squares) and of 27 at optimised conditions (white circles to black circles). This gave a best GFP yield of 3.7 g from a 3 L culture. In both cases, the effective induction period was 48 h in contrast to 8 h under standard (white squares) or DoE-optimised (white circles) conditions, or 15 h when only the glycerol concentration was increased (to 40 g L^-1^; grey circles). Figure 5 (inset) confirms that the observed improvement in yield during a mixed feed induction is not simply due to improved biomass accumulation as the corresponding improvements in specific yield are by a factor of 3.3 and 7.5, respectively. Rather it suggests a better matching of the induction regime to the requirements of the cellular metabolism under the specific DoE conditions of 21.5°C, pH 7.6, 90% DO used here. This results in a substantial yield improvement.

**Figure 7:**
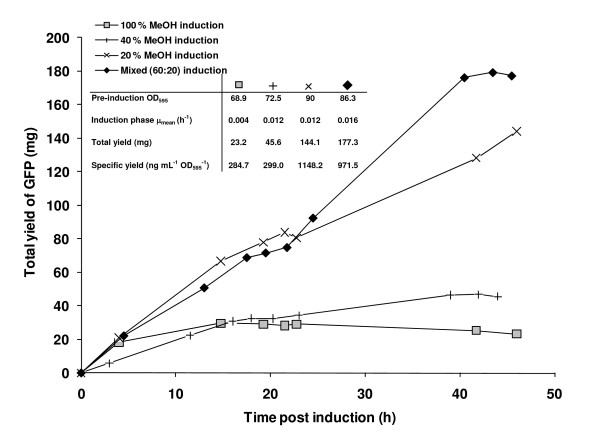
**Total GFP yields over a 48 h induction period in 1 L fed-batch cultures**. The effect of the following induction regimes on cultures grown under standard production conditions (30°C, pH 6.0, 30% DO) and 40 g L^-1 ^glycerol prior to induction are shown: 100% methanol (grey squares); 40% methanol (crosses); 20% methanol (diagonal crosses) and a mixed 60% methanol, 20% sorbitol regime (black diamonds).

**Figure 8:**
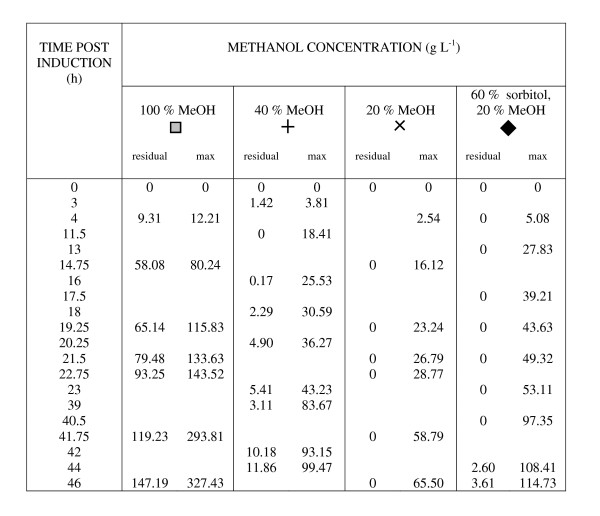
**Analysis of residual methanol concentrations from cultures in Figure 7**. Residual methanol concentrations are shown in g/L for cultures grown under standard conditions (30°C, pH6, 30% DO) with 40 g/L glycerol and the indicated induction regimes. The symbols used in Figure 7 are also shown in each case for ease of comparison between Figure 7 and Figure 8. The theoretical maximum methanol concentration (if none had been metabolised; 'max') is presented for comparison.

The methanol analysis in Figure 6 shows that on moving to a mixed induction that the methanol was fully utilised by the cells under both standard and DoE-optimised conditions (black symbols). These cells also had increased mean specific growth rates in line with previously-published values [25]. It appeared that the methanol concentration was limiting for the cells grown under standard conditions (black squares), contributing to the lower specific yield in those cells compared to ones grown under DoE-optimised conditions (black circles). Further investigation of the induction regime in 1 L bioreactor cultures showed that reducing the feed to 20% or 10% methanol gave no yield improvement over 100% methanol for DoE-optimised conditions (data not shown), but that some yield improvement could be achieved by reducing the feed to 20% methanol in both the absence or presence of sorbitol for cells grown under standard conditions. Figure 7 shows that the effective induction period was increased to 48 h in these cases and is supported by the methanol data in Figure 8.

## Discussion

In an attempt to rationalise recombinant protein production, DoE has been implemented by a number of groups to examine the influence of selected input parameters on yield [8-11]. One example investigated the yield of a therapeutic F_c _fusion protein produced in *P. pastoris *as a function of pH, T, salt supplementation and the glycerol concentration in the pre-induction medium [7]. Not surprisingly, the authors concluded that there is unlikely to be a single set of culture conditions that will work well for all types of proteins and therefore that thorough process development will be required on a case-by-case basis for large-scale production in *P. pastoris *to be feasible.

The challenge, then, was to design a system that efficiently screens for optimal production conditions in a medium- to high-throughput format, and that can be scaled to larger vessels. In order to bridge the gap between screening and production, we examined whether it would be possible to use DoE to efficiently model the yield of a secreted recombinant protein in 6 mL *P. pastoris *cultures grown in an M24, and in particular whether the model would be scalable to 3 L cultures when grown in a bioreactor. The results presented here show that a predictive model based on specific yield achieves these objectives. This relies on the fact that the M24 is capable of tight control of culture parameters in contrast to cultures grown in shake flasks. Since shake flask-grown cultures suffer from heterogeneous oxygen transfer [26] they cannot be used as reliable indicators of bioreactor yields (Figure 3). This observation backs up much anecdotal evidence, as well as specific examples in the literature, that on moving from shake flask to bioreactor there is no guarantee of scalability [27,28]. Indeed by screening in shake flasks, or uncontrolled multi-well plates, conditions appropriate for scale-up might be missed. Notwithstanding this, in trials where investigators are limited to working in shake flasks, it is clear that spending time optimising appropriate combinations of culture pH, T and DO (through changing shaking speeds) is likely to have benefits in improving total yields. Furthermore, examining media with increased glycerol concentrations and varying induction protocols should be targeted for optimisation.

We noted that while a model of typically-varied culture parameters (pH, T, DO) was predictive and scalable, the fact that it was based on yields normalised for culture volume and biomass suggested that further improvements would be possible by boosting the pre-induction biomass itself. Pre-induction glycerol-feeding schemes have previously been shown to have a significant effect on the post-induction production of a recombinant growth hormone [29] and we noted that this was also the case for our cultures. Moreover, while our protein yields reached a plateau 15 h post-induction when grown on 40 g L^-1 ^glycerol in contrast to 8 h on 10 g L^-1^, there was still room for further improvement. In the same study on growth hormone production [29], mixed feeding of methanol and glycerol during induction further improved specific production rates following induction of high density cultures (320 g L^-1^) which continued to accumulate biomass to a final cell density of 428 g L^-1^. Our methanol data for GFP-producing cells (Figures 6 and 8) support this view.

The specific advantages of mixed feeds for the production of recombinant proteins by *P. pastoris *were recently analysed quantitatively [23,24]. The authors of these studies concluded that replacing methanol as sole carbon source improved productivity due to increased biomass yields during mixed substrate growth, with 43% methanol and 57% sorbitol being optimal for the production of recombinant avidin. We noted that the effective induction period of cultures induced with both methanol and sorbitol increased to the full 48 h of the induction period (Figure 5). Figure 5 shows that this total yield increase is not solely due to increasing biomass throughout the induction phase. We suggest that matching the induction regime with the metabolism of the producing cells, which we have previously highlighted as a key indicator of production efficiency [30], is an area requiring closer examination, and would benefit from a targeted DoE approach to build on the model presented here.

## Conclusion

In summary, we present a strategy for optimising specific yield in the induction phase through DoE. Our methodology of using small-scale cultures to rapidly produce a scalable, predictive model should be transferable to other target proteins and host systems.

## Competing interests

The authors declare that they have no competing interests.

## Authors' contributions

WH and RS were involved in all aspects of the experimental design, data collection, analysis and interpretation. RD assisted with the microbial cultures, and focused particularly on the methanol analyses. MW performed the statistical analysis of the model. RB directed the study, co-ordinated the data analysis and interpretation, and drafted the manuscript. All authors contributed to, read and approved the final version of the manuscript.

## Supplementary Material

Additional file 1 **Results of the first round of DoE**. The data provided summarise the first model building experiment. Click here for file
