# Supplementary material for: Developing a scalable model of recombinant protein yield from Pichia pastoris: the influence of culture conditions, biomass and induction regime
Source: Microb Cell Fact. 2009 Jul 1;8:35. doi: 10.1186/1475-2859-8-35 (PMC2717918; doi:10.1186/1475-2859-8-35)
Supplement: Additional file 1 — Results of the first round of DoE. The data provided summarise the first model building experiment. [file 1475-2859-8-35-S1.pdf]

Additional File 1

In selecting the input parameters used in this study, we were guided by the results of our first DoE. To make the logic of our strategy clearer, we refer to the results of this first DoE, here in additional file 1. The input parameters shown in Table A1 were selected. The lowest pH that was compatible with the limits of the M24 was 5.3, allowing us to explore a range from 5.3 to 6.7. In this design, we also examined the optimal growth temperature of 30 °C. Relative fluorescent units (RFU) and the optical density at 595 nm (OD_{595}) were measured in triplicate 48 h post induction and used to build a first model based on RFU normalised to OD_{595} (Equation A1).

Equation A1: $RFU\ OD_{595}^{-1} = 853.114 + (19.9937 \times T) - (447.489 \times pH) + (2.248 \times DO) + (50.5565 \times pH^2) - (3.15409 \times T \times pH) - (0.32396 \times pH \times DO)$

Table A2 summarises the statistical assessment of the model by analysis of variance (ANOVA). Regression analysis of the model indicated that it explains 89 % of the response variation.

It was clear from this first DoE that the resultant model did not capture the optimised set of conditions for maximal protein yield. However, the model did suggest that the design space should specifically examine higher pH levels, and lower temperatures in a follow-up DoE. This is the DoE presented in the manuscript.

Table A1. Specification of the input factors and measurable outputs for the model building experiments.

The input factors were temperature (T), pH and % dissolved oxygen (DO).

CODED FACTORS			INPUT FACTORS		
T	pH	DO	T (°C)	pH	DO (%)
-1	1	0	20	6.7	45
-1	-1	0	20	5.3	45
-1	0	1	20	6	80
-1	0	-1	20	6	10
0	1	-1	25	6.7	10
0	0	1	25	6	80
0	0	-1	25	6	10
0	1	1	25	6.7	80
0	0	0	25	6	45
0	-1	-1	25	5.3	10
0	-1	1	25	5.3	80
1	1	0	30	6.7	45
1	0	-1	30	6	10
1	0	1	30	6	80
1	-1	0	30	5.3	45

Table A2. Statistical significance of the predictive model by analysis of variance (ANOVA).

The statistical significance of the relationship between the predictors and the response of the model was assessed using analysis of variance (ANOVA), which employs Fisher's *F*-test. The goodness of fit of the model is 89 %, as determined by the quotient of residual sum of squares/total sum of squares (R^2 = 0.89).

Source	Degrees of Freedom	Sum of Squares	Mean Square	*F* statistic	*p* value
Regression	6	21343.9	3557.32	11.09	0.002
Linear	3	18313.4	6104.46	19.03	0.001
Square	1	2291.1	2291.09	7.14	0.028
Interaction	2	739.5	369.73	1.15	0.363
Residual	8	2566.4	320.80		
Total	14	23910.3			
